# Establishing the Neck Disability Index as a Valid Tool for Assessing Persistent Neck Pain in the Albanian Population

**DOI:** 10.3390/medicina61060955

**Published:** 2025-05-22

**Authors:** Elda Zeqiri, Erda Qorri, Jasemin Todri, Orges Lena

**Affiliations:** 1 Health Science PhD Program, UCAM Universidad Católica San Antonio de Murcia, Campus de los Jerónimos, Nº 135 Guadalupe, 30107 Murcia, Spain; elda_zeqiri@gmx.de; 2 Dentistry Faculty, Albanian University, 1001 Tirana, Albania; e.qorri@albanianuniversity.edu.al; 3 ÍTEM—Innovation in Manual and Physical Therapies, Research Group, Physiotherapy Department, UCAM Universidad Católica San Antonio de Murcia, Campus de los Jerónimos, Nº 135 Guadalupe, 30107 Murcia, Spain; lorges@ucam.edu

**Keywords:** Albanian Neck Disability Index, translation, validation, measurement tool, neck pain

## Abstract

*Background and Objectives:* The Neck Disability Index (NDI) is one of the most widely used instruments for assessing self-reported neck-related functional limitations. However, a validated Albanian version has not previously existed, limiting its application in Albanian-speaking populations. This study aimed to translate, culturally adapt, and evaluate the psychometric properties of the Albanian version of the NDI (ANDI), focusing on its reliability and internal consistency. *Materials and Methods:* A standard forward–backward translation methodology was used to develop the Albanian version of the NDI, followed by cultural adaptation. A total of 83 participants with neck pain completed the ANDI at two time points, three days apart. Test–retest reliability was assessed using the intraclass correlation coefficient (ICC), while internal consistency was evaluated using Cronbach’s alpha and the item–total correlation (ITC). *Results:* The ICC values for all 10 sections of the ANDI ranged from 0.95 to 0.99, indicating excellent test–retest reliability. The highest reliability was observed in (personal care) and (pain intensity) with ICCs of 0.99 and 0.98, respectively. All sections demonstrated strong internal consistency, with ITC values ranging from 0.91 to 0.98 and a Cronbach’s alpha of 0.96. Mean scores between test sessions showed negligible variation, further confirming score stability. *Conclusions:* The Albanian version of the NDI demonstrated excellent reliability and internal consistency, confirming its validity for use in clinical and research settings. This represents the first validated neck disability assessment tool for the Albanian-speaking population, supporting more inclusive musculoskeletal health assessment.

## 1. Introduction

Neck pain is a prevalent musculoskeletal condition affecting a significant portion of the global population, often leading to disability and reduced quality of life [1]. Assessing the functional limitations associated with neck pain is essential for clinical decision-making, treatment planning, and evaluating therapeutic outcomes [2]. Among the various assessment tools available, the **Neck Disability Index (NDI)** is one of the most widely used and validated patient-reported outcome measures for neck-related disability [3,4]. The NDI, developed by Vernon and Mior in 1991, is a 10-item questionnaire designed to evaluate the impact of neck pain on daily activities, including personal care, lifting, reading, work, driving, sleeping, recreation, and concentration [5]. Each item is scored on a scale from 0 to 5, with higher scores indicating greater disability. Given its widespread application in both clinical and research settings, it is crucial to establish the psychometric properties of the NDI, particularly its **validity and reliability**.

Cross-cultural adaptation of the NDI is critical for ensuring its relevance and accuracy across different linguistic and cultural contexts. Previous studies have demonstrated the importance of this process in various languages. Santiago-Reynoso et al. (2021) conducted a transcultural adaptation and psychometric evaluation of the Mexican Spanish NDI [6]. The study confirmed its good internal consistency (Cronbach’s alpha = 0.85) and test–retest reliability (ICC = 0.86), ensuring its validity for Spanish-speaking populations [6]. Similarly, Koh & Koh (2022) adapted and validated the Malay version of the NDI, reporting excellent internal consistency (Cronbach’s alpha = 0.84) and strong construct validity through correlations with the Visual Analog Scale (VAS) [7]. A 2023 study assessed the psychometric properties of the Hindi version of the NDI in patients with chronic neck pain. The translation followed international guidelines, demonstrating strong reliability (ICC = 0.92) and high construct validity [8]. Lim et al. (2020) validated the simplified Chinese version of the NDI, highlighting its excellent internal consistency (Cronbach’s alpha = 0.92) and responsiveness in patients undergoing rehabilitation for neck pain [9]. Shashua et al. (2016) translated and validated the Hebrew NDI, emphasizing linguistic and cultural equivalence [10]. The study reported a Cronbach’s alpha between 0.85 and 0.92 and a significant correlation with the neck pain rating scale (NPRS) and patient-specific functional scale (PSFS) [10]. Farooq et al. (2024) examined the reliability and validity of the Urdu version of the NDI [11]. The study found high internal consistency (Cronbach’s alpha = 0.96) and excellent test–retest reliability (ICC = 0.92), ensuring its applicability in Urdu-speaking populations [11]. Lauridsen et al. (2017) provided insights into the Danish NDI’s factor structure, generalizability, and responsiveness [12]. Their findings suggested a one-factor model for improved interpretability and strong internal consistency (Cronbach’s alpha = 0.89) [12]. Table 1 gives a detailed comparison of the validity and reliability of the NDI for the different language versions in which it is already used [6,7,8,9,10,11,12,13,14,15,16,17,18].

Additionally, Saltychev et al. (2024) conducted a systematic review and meta-analysis, reinforcing the NDI’s global validity and reliability across multiple languages and populations [19]. Their review highlighted the robustness of the NDI’s validity and reliability across different cultural settings, with pooled Cronbach’s alpha values ranging from 0.80 to 0.90 [19].

Although the NDI is the most widely used and validated instrument for assessing disability due to neck pain across various languages and cultural settings, it is not the only available tool. Several other clinometric scales have been developed to capture different aspects of neck-related disability. The Neck Pain and Disability Scale (NPAD), for example, provides a comprehensive 20-item visual analog scale covering pain intensity, emotional effects, and daily functioning, offering a broader assessment of the psychosocial impacts of neck pain [20]. Similarly, the Neck Bournemouth Questionnaire (NBQ) integrates cognitive and affective dimensions such as anxiety and depression, using a biopsychosocial model particularly relevant in manual therapy and chiropractic contexts [21]. The QuickDASH, a shortened version of the Disabilities of the Arm, Shoulder, and Hand questionnaire, while not neck-specific, is frequently used in clinical practice for patients with upper extremity or cervical spine disorders due to its brevity and ease of use [22]. Despite the availability of these alternative instruments, the NDI remains the most suitable choice for cross-cultural adaptation into Albanian due to its extensive international use, strong psychometric properties, concise format, and unidimensional structure, which simplify both clinical implementation and research comparability [6,7]. The present study addresses the need for a validated Albanian version of the NDI to improve the assessment of neck-related disability in Albanian-speaking populations. In this context, translating and cross-culturally adapting the NDI for the Albanian population with neck pain is essential to ensure its applicability and accuracy in this demographic. Language and cultural differences can significantly impact how individuals interpret and respond to health questionnaires, necessitating a rigorous adaptation process to maintain conceptual equivalence. This article aims to detail the translation, cultural adaptation, and validation of the NDI for the first time in the Albanian population. By following established guidelines for cross-cultural adaptation, we seek to ensure that the Albanian version of the NDI retains its psychometric integrity and clinical utility. Understanding these adaptations will be instrumental for clinicians and researchers working with Albanian-speaking individuals suffering from neck pain.

## 2. Materials and Methods

### 2.1. Study Design

This prospective, multi-center study sequentially enrolled adult volunteers between the ages of 18 and 80 years who were native Albanian speakers. Participants were recruited from outpatient clinics and local community health centers. The study received ethical approval from the Albanian Health and Social Ministry Ethics Committee (Protocol ID: 208/9) and from the Ethics Committee of the Catholic University of Murcia (UCAM) (Approval ID: CE012516). It was prospectively registered at ClinicalTrials.gov under the identifier NCT06834048. All participants were healthy individuals without significant neck pathology, ensuring an appropriate sample for initial validation. The study was conducted in accordance with the principles outlined in the Declaration of Helsinki, and informed consent was obtained from all participants. The MAPI Research Trust, copyright owner of the NDI, allowed the translation into Albanian (Supplementary Materials).

### 2.2. Translation and Cross-Cultural Adaptation

The translation and cross-cultural adaptation of the Neck Disability Index (NDI) followed internationally recommended guidelines, including those described by Tsang et al. (2017) and the methodology proposed by Beaton et al. [23,24]. The process included the following steps [23,24,25]:

1. Forward Translation: Two independent bilingual translators, whose native language was Albanian and who were fluent in English, translated the original English version of the NDI into Albanian.
2. Synthesis: A consensus version of the forward translations was synthesized by the translators and a third reviewer.
3. Backward Translation: Two different bilingual translators, blinded to the original version and with no medical background, independently translated the synthesized Albanian version back into English.
4. Expert Committee Review: An expert committee composed of translators, physiotherapists, linguists, and methodologists reviewed all versions to achieve semantic, idiomatic, experiential, and conceptual equivalence.
5. Pre-testing: The pre-final version was administered to a pilot group of 83 native Albanian speakers. Participants were asked to provide feedback regarding clarity, relevance, and comprehensibility of the items. Necessary modifications were made based on their input.

**Figure 1:**
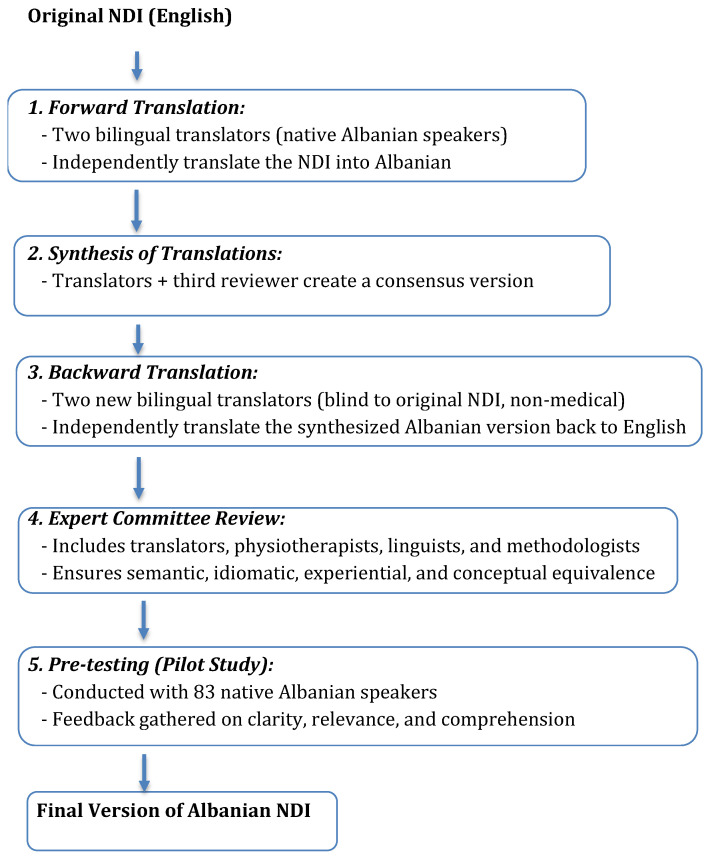
Flow diagram of Albanian NDI translation and cross-cultural adaptation process.

The following diagram illustrates the translation and cross-cultural adaptation process of NDI into Albanian (Figure 1).

### 2.3. Participants

The validation sample included adult native Albanian speakers aged 18–80 years. Participants were recruited from both urban and rural regions to ensure linguistic and cultural diversity. Inclusion criteria required individuals to be experiencing non-traumatic, primarily mechanical neck pain of at least 4 weeks’ duration, which qualifies as subacute to chronic, and be functionally independent and cognitively able to complete the questionnaire in Albanian. Patients with recent traumatic injuries, surgical history, or neurological, psychiatric, or systemic musculoskeletal conditions were excluded to reduce clinical confounding.

### 2.4. Instrument: Neck Disability Index (NDI)

The NDI is a widely used, self-administered questionnaire designed to measure neck-specific disability. It consists of 10 items, each scored on a 6-point scale (0–5), assessing pain and the degree to which neck problems affect daily activities, including personal care, lifting, reading, work, driving, sleeping, and recreation. The total score ranges from 0 to 50, with higher scores indicating greater disability [5].

### 2.5. Procedure

Participants (*n* = 83) completed the Albanian version of the NDI (ANDI) twice within a 3-day interval to assess test–retest reliability. Demographic data were collected, and clinical variables were documented when applicable. This sample size was determined with reference to established psychometric guidelines for validation studies. According to Kyriazos (2018), for robust factor analysis and psychometric evaluation, a minimum subject-to-item ratio of 5:1 is typically recommended, with a preferred ratio of 7:1 or higher for increased stability [26]. Given that the NDI contains 10 items, a sample size of 83 exceeds the 7:1 ratio, satisfying these recommendations for adequate power and reliability in exploratory or confirmatory factor analysis.

### 2.6. Psychometric Testing

The psychometric evaluation included an assessment of internal consistency, content validity, and test–retest reliability. Internal consistency was evaluated using Cronbach’s alpha coefficient, a statistical measure that examines the degree of interrelatedness among the items in a scale. Test–retest reliability was examined using the intraclass correlation coefficient (ICC), which assesses the stability of the questionnaire.

### 2.7. Data Analysis

Descriptive statistics were used to summarize participant demographics and NDI scores. Internal consistency was considered acceptable with Cronbach’s alpha ≥ 0.70. ICC values ≥ 0.75 indicated good test–retest reliability. Validity was examined using Spearman’s correlation coefficients, comparing the NDI scores with participant-reported health status. All statistical analyses were performed using SPSS version 25, with significance set at *p* < 0.05.

## 3. Results

Table 2 presents the descriptive characteristics of the sample used in the ANDI validation study. A total of 83 participants were included. The mean age of the participants was 41.75 years (SD = 14.99), suggesting a sample primarily composed of adults in early to middle adulthood. The participants had a mean weight of 72.43 kg (SD = 14.09) and a mean height of 171.41 cm (SD = 8.81), indicating a relatively average body composition across the sample. In terms of gender distribution, 47 participants were female (56.6%) and 36 were male (43.4%), representing a balanced sample with a slight predominance of women. Regarding educational background, the majority of participants had completed higher graduation (*n* = 47, 56.6%), followed by those with postgraduate education (*n* = 30, 36.1%). A smaller part of the sample held a PhD (*n* = 4, 4.8%), while only 2 participants (2.4%) had a middle-level education. The participants came from diverse professional backgrounds. The most common profession was categorized as “Other” (*n* = 29, 34.94%), indicating a wide range of occupations. Among the “Other” occupations, the most frequently reported was Jurist (*n* = 5), followed by Physiotherapist (*n* = 4), and both Doctor and Dentist with 3 participants each. Several professions were represented by two participants each, including Teacher and Translator, while the remaining occupations had only one participant each, highlighting even greater variety. These include Laboratory Technician, Cashier, Accountant, Bank Teller, Salesperson, Veterinarian, Driver, Electrician, Mechanic, and Hairdresser. The remaining participants included Economists (*n* = 14, 16.9%), Students (*n* = 13, 15.7%), Nurses and Secretaries (each *n* = 7, 8.4%), and Professors (*n* = 6, 7.2%) (Table 2).

Table 3 presents the results of a factor analysis conducted to examine the construct validity of the ANDI. The Kaiser–Meyer–Olkin (KMO) Measure of Sampling Adequacy is reported at 0.91, which is considered excellent, indicating that the sample size is adequate and appropriate for factor analysis. Additionally, Bartlett’s Test of Sphericity yielded a value of 516.51 with a significance level (Sig.) of 0.000, confirming that the correlations among the items are sufficiently strong for factor analysis.

A Principal Component Analysis (PCA) was conducted, and a single-factor solution was extracted. The factor loadings for all 10 sections of the ANDI ranged from 0.75 to 0.96, indicating strong correlations between each item and the extracted factor. Specifically, Section 3 had the highest loading (0.96), showing it is most strongly associated with the underlying factor. Section 10 had the lowest loading (0.75), but it is still well above the commonly accepted minimum threshold of 0.40, indicating a good contribution to the factor. Most items, such as Sections 1, 2, 4 and 5, also show high factor loadings (above 0.85), reinforcing the unidimensionality of the scale.

Table 4 reports on the test–retest reliability and internal consistency of the ANDI across two measurements taken from the same group of 83 participants. The table evaluates 10 sections of the index, each reflecting different aspects of neck disability. In terms of test–retest reliability, the ICC values for all sections ranged from 0.95 to 0.99, which were considered excellent. Typically, an ICC value above 0.75 indicates good reliability, while values above 0.90 reflect exceptionally high stability over time. Section 2 (personal care) and Section 1 (pain intensity) showed ICCs of 0.99 and 0.98, respectively, indicating highly consistent results over time. The lowest ICC was observed in Section 3 (lifting), with a value of 0.95, still reflecting strong test–retest agreement even for more physically demanding tasks. Regarding internal consistency, all sections reported item–total correlation (ITC) values between 0.91 and 0.98, suggesting that each item strongly correlated with the overall scale. This indicated that each question contributed significantly to assessing neck disability. The Cronbach’s alpha for each section was 0.96, demonstrating very high internal consistency throughout the index. Mean scores across the two time points remained remarkably consistent, with only minimal differences observed between the first and second measurements, further reinforcing the tool’s reliability. Section 5 (headaches) had one of the highest scores (2.05 ± 1.14 at Time 1), while Section 2 (personal care) reflected lower difficulty levels (1.54 ± 1.33).

## 4. Discussion

The present study evaluated the psychometric properties of the ANDI, focusing on its cross-cultural adaptation, construct validity, internal consistency, and test–retest reliability. Our findings affirm that the ANDI is not only a reliable and valid tool for assessing neck-related disability but also one that performs strongly across all psychometric domains. The demographic profile of our participants, who were relatively well educated, diverse in profession, and functionally independent, likely contributed to the clarity and consistency with which the instrument was completed. This enhances confidence in the generalizability of the results across varying age groups, educational levels, and occupational backgrounds.

Notably, the test–retest reliability results were exceptionally high, with an ICC ranging from 0.95 to 0.99, surpassing those reported in many other validated versions of the NDI. While ICCs above 0.90 are generally classified as excellent, our consistently higher values may reflect several influencing factors. One possibility is the clarity and semantic precision achieved during the translation and cultural adaptation process, guided by Beaton et al.’s rigorous methodology [24]. The pilot testing phase provided feedback that allowed for refinement of item wording, potentially reducing variability in interpretation. Another contributing factor may be the homogeneity of the sample in terms of cognitive and language comprehension, as participants were screened to ensure fluency in Albanian and sufficient cognitive ability to engage with the tool. Compared to versions developed for more heterogeneous or multilingual populations such as the German (ICC 0.88–0.98) [13] or Japanese (ICC up to 0.94) [14], the Albanian cohort may have provided a more stable response base, thus explaining the tighter reliability range. Comparable values have been noted in Hindi [15], Serbian [16], and Malay [7] versions, demonstrating the NDI’s consistency across diverse cultural and linguistic settings.

Similarly, the internal consistency of the ANDI was outstanding, with ITC values between 0.91 and 0.98, and a Cronbach’s alpha of 0.96. These results suggest a high degree of coherence among the scale’s items, further supported by our factor analysis findings. The strong internal consistency not only confirms that the instrument reliably measures the intended construct but also suggests minimal redundancy among items, a particularly valuable feature in clinical settings where time efficiency and interpretive clarity are essential. These values exceed those reported in other cross-cultural adaptations, such as the Dutch version (α = 0.87) [17] and the French Total Disability Index (α = 0.93) [27], indicating that the ANDI performs at the upper limit of psychometric quality.

The stability of mean scores across both measurement time points adds another layer of evidence supporting the ANDI’s reliability. The minimal variations observed underscore its resilience to recall bias or temporary fluctuations in symptom perception. This level of temporal stability is particularly important in longitudinal research and clinical monitoring, where consistency over time is crucial for detecting meaningful changes.

Moreover, systematic reviews on cross-cultural adaptations [28] emphasize the need for translated tools to maintain reliability, internal consistency, and cultural relevance. The ANDI appears to fulfill all three. Its performance in this study positions it as a robust alternative to the original English version and aligns well with validated tools in other languages [18,28,29]. Importantly, our findings also highlight the broader implications of cultural adaptation not simply translating words but ensuring conceptual and experiential equivalence [23,24]. This holistic approach to adaptation likely contributed to the instrument’s psychometric strength and underscores the value of culturally nuanced validation studies.

In summary, the slightly higher ICC and internal consistency values observed in the Albanian version of the NDI may reflect a combination of methodological rigor, sample characteristics, and cultural–linguistic clarity factors that collectively enhance its utility as a reliable measure for clinical and research applications in Albanian-speaking populations.

### 4.1. Implications for Practice and Research

The high reliability and internal consistency of the ANDI have important implications for both clinical practice and research. Clinicians can confidently use the ANDI to assess neck disability in Albanian-speaking patients, supporting accurate diagnosis, treatment planning, and progress monitoring. Additionally, the tool can facilitate cross-cultural research by providing a validated outcome measure that aligns methodologically with those used in other regions.

Given the increasing prevalence of neck pain globally and in Eastern European contexts [30,31,32], the availability of a validated Albanian measure is timely. This study represents the first translation, cultural adaptation, and psychometric validation of the Neck Disability Index for the Albanian-speaking population. Until now, clinicians and researchers working with Albanian patients lacked an evidence-based, linguistically, and culturally appropriate tool for assessing neck disability. The development of the ANDI fills this critical gap, enabling standardized evaluation of neck-related functional impairment in both clinical and research settings. This advancement not only enhances the quality of musculoskeletal care in Albanian-speaking regions but also lays the groundwork for future comparative studies and cross-cultural research involving diverse populations.

### 4.2. Limitations and Future Directions

While the current findings are promising, a few limitations should be acknowledged. First, the sample was limited to a single cohort of 83 participants, which may affect the generalizability of results. Future research should aim to validate the ANDI across broader demographic and clinical subgroups, including older adults and individuals with specific cervical pathologies. Although this study assessed internal consistency and test–retest reliability, it did not evaluate responsiveness to clinical change, a critical psychometric property for longitudinal applications. Responsiveness indicates the instrument’s sensitivity to detecting meaningful changes over time, such as those resulting from therapeutic interventions. The absence of responsiveness data limits the current application of the ANDI in monitoring treatment outcomes and should be explicitly addressed in future validation work through prospective, intervention-based study designs.

Moreover, the construct validity of the ANDI—its ability to measure the theoretical concept of neck-related disability—was not explored using convergent or divergent validity testing against other established instruments as NPAD, NBQ, or QuickDASH. Including such comparative analyses would strengthen the interpretability of ANDI scores and clarify its position within the broader family of neck disability measurement tools.

Another avenue for improvement is the integration of qualitative patient feedback to assess item clarity, linguistic appropriateness, and cultural relevance. This approach would help identify nuances that quantitative psychometric evaluations may overlook and contribute to iterative refinement of the scale.

Finally, future research could explore the cross-cultural comparability of the ANDI with versions in other languages using differential item functioning or item response theory models. This would allow researchers to better understand how different populations interpret and respond to similar items, paving the way for internationally harmonized disability assessments.

## 5. Conclusions

The ANDI demonstrated excellent test–retest reliability and internal consistency. These results support its use as a valid and reliable tool for assessing neck-related disability in Albanian-speaking populations, contributing to improved clinical care and research quality in this linguistic and cultural context. While the current validation offers an important first step, future studies should build on this foundation by broadening participant diversity, incorporating responsiveness and construct validity analyses, and exploring both qualitative insights and cross-national comparisons.

## Figures and Tables

**Table 1:** Reliability and validity of the Neck Disability Index (NDI) for the different language versions.

NDI Language	Authors (Year)	Cronbach’s Alpha	ICC	Population
Mexican Spanish	Santiago-Reynoso et al. (2021) [6]	0.91	0.97	Chronic neck pain patients
Malay	Koh & Koh (2022) [7]	0.89	0.96	Multilingual urban population
Hindi	Geete et al. (2023) [8]	0.9	0.96	Indian patients with neck pain
Simplified Chinese	Yang et al. (2020) [9]	0.88	0.93	Chinese population
Hebrew	Shashua et al. (2016) [10]	0.89	0.95	Israeli patients
Urdu	Farooq et al. (2024) [11]	0.88	0.93	Pakistani patients
Danish	Lauridsen et al. (2017) [12]	0.92	0.94	General Danish population
German	Swanenburg et al. (2014) [13]	0.92	0.96	Rehabilitation patients
Japanese	Takeshita et al. (2013) [14]	0.88	0.9	Japanese patients with neck pain
Hindi (rural)	Sidiq et al. (2024) [15]	0.91	0.94	Rural population, northern India
Serbian	Jovicic et al. (2018) [16]	0.88	0.95	Cervical radiculopathy patients
Dutch	Jorritsma et al. (2012) [17]	0.9	0.94	Dutch-speaking patients
Turkish	Bicer et al. (2004) [18]	0.88	0.93	Turkish patients with chronic pain

NDI: Neck Disability Index.

**Table 2:** Descriptive characteristics of the sample.

Participants Characteristics		N	Mean	SD
Age		83	41.75	14.99
Weight		83	72.43	14.09
Height		83	171.41	8.81
Gender	Female	47		
	Male	36		
Study level	Middle level	2		
	Higher graduation	47		
	Postgraduation	30		
	PhD	4		
Profession	Economist	14		
	Student	13		
	Nurse	7		
	Retired	7		
	Secretary	7		
	Professor	6		
	Other	36		

ANDI: Albanian version of Neck Disability Index (NDI); N: number of participants; SD: standard deviation.

**Table 3:** Factor loading values for single-factor solution of ANDI.

ANDI Section Analysis	Factor 1
Kaiser–Meyer–Olkin Measure of Sampling Adequacy	0.91
Bartlett’s Test of Sphericity	516.51
Sig.	0.00
Section 1	0.86
Section 2	0.87
Section 3	0.96
Section 4	0.90
Section 5	0.91
Section 6	0.80
Section 7	0.82
Section 8	0.77
Section 9	0.87
Section 10	0.75

ANDI: Albanian version of Neck Disability Index (NDI).

**Table 4:** Mean and reliability results of ANDI.

NDI	ANDI	1st Measurement (N = 83) Mean ± SD	2nd Measurement (N = 83) Mean ± SD	ICC	95% CI	ITC	Cronbach’s Alpha
Section 1—PAIN INTENSITY	INTENSITETI I DHIMBJES	1.72 ± 1.15	1.72 ± 1.06	0.98	0.97–0.99	0.96	0.96
Section 2—PERSONAL CARE	KUJDESI PERSONAL	1.54 ± 1.33	1.55 ± 1.29	0.99	0.97–0.99	0.98	0.96
Section 3—LIFTING	NGRITJA	1.63 ± 1.07	1.64 ± 0.98	0.95	0.93–0.97	0.91	0.96
Section 4—READING	LEXIMI	1.88 ± 1.07	1.87 ± 1.03	0.98	0.97–0.99	0.96	0.96
Section 5—HEADACHES	DHIMBJA E KOKËS	2.05 ± 1.14	2.01 ± 1.05	0.97	0.97–0.98	0.95	0.96
Section 6—CONCENTRATION	PËRQËNDRIMI	1.93 ± 1.23	1.82 ± 1.20	0.96	0.94–0.98	0.93	0.96
Section 7—WORK	PUNA	1.72 ± 1.27	1.70 ± 1.15	0.97	0.95–0.98	0.95	0.96
Section 8—DRIVING	DREJTIMI I MAKINËS	1.83 ± 1.32	1.83 ± 1.21	0.97	0.95–0.99	0.94	0.96
Section 9—SLEEPING	GJUMI	1.87 ± 1.28	1.80 ± 1.19	0.97	0.95–0.100	0.94	0.96
Section 10—RECREATION	RECREACIONI	1.78 ± 1.18	1.75 ± 1.06	0.97	0.95–0.98	0.94	0.96

ANDI: Albanian version of Neck Disability Index (NDI); N: number of participants; SD: standard deviation; ICC: interclass correlation coefficient; CI: confidence interval; C: item–total correlation.

## Data Availability

The original contributions presented in this study are included in the article. Further inquiries can be directed to the corresponding author.

## Supplementary Materials

The following supporting information can be downloaded at https://www.mdpi.com/article/10.3390/medicina61060955/s1, File S1: Indeksi i kufizuar i qafes.
