# Supplementary material for: Establishing the Neck Disability Index as a Valid Tool for Assessing Persistent Neck Pain in the Albanian Population
Source: Medicina (Kaunas). 2025 May 22;61(6):955. doi: 10.3390/medicina61060955 (PMC12195372; doi:10.3390/medicina61060955)
Supplement: Supplementary file 1 [file medicina-61-00955-s001.zip › medicina-3628290-supplementary.pdf]

Indeksi i kufizuar i qafes ©

KY PYETËSOR ËSHTË KRIJUAR PËR TË NA INFORMUAR SE SI **DHIMBJA E QAFËS** KA NDIKUAR NË AFTËSINË TUAJ PËR TË MENAXHUAR JETËN E PËRDITSHME. JU LUTEMI PËRGJIGJUNI ÇDO SEKSIONI DHE ZGJIDHNI NË ÇDO SEKSION VETËM **NJË KUTI** QË PËRSHTATET PËR JU.
E KUPTOJMË SE MUND TË KONSIDERONI SE DY OSE MË SHUMË OPSIONE NË ÇDO SEKSION MUND TË PËRSHTATEN PËR JU, POR JU LUTEMI ZGJIDHNI VETËM NJË KUTI QË ËSHTË **MË E AFËRT** ME PROBLEMIN TUAJ.

SEKSIONI 1 - INTENSITETI I DHIMBJES

- ☐ Nuk kam dhimbje për momentin
- ☐ Dhimbja është shumë e lehtë për momentin
- ☐ Dhimbja është e moderuar për momentin
- ☐ Dhimbja është mjaft e fortë për momentin
- ☐ Dhimbja është shumë e fortë për momentin
- ☐ Dhimbja është më e keqja që mund të imagjinohet për momentin

SEKSIONI 2 - KUJDESI PERSONAL

- ☐ Unë mund të kujdesem për veten normalisht pa pasur dhimbje shtesë
- ☐ Unë mund të kujdesem për veten normalisht, por kjo shkakton dhimbje shtesë
- ☐ Kam dhimbje kur kujdesem për veten dhe jam i ngadaltë dhe i kujdesshëm
- ☐ Kam nevojë për ndihmë, por mund të menaxhoj pjesën më të madhe të kujdesit tim personal
- ☐ Kam çdo ditë nevojë për ndihmë në shumicën e aspekteve të kujdesit për veten
- ☐ Nuk vishem dot, lahem me vështirësi dhe rri në shtrat

SEKSIONI 3 - NGRITJA

- ☐ Mund të ngre pesha të rënda pa dhimbje shtesë
- ☐ Mund të ngre pesha të rënda, por kjo sjell dhimbje shtesë
- ☐ Dhimbja më pengon të ngre pesha të rënda nga dyshemeja, por mund ta përballoj nëse pozicionohen në mënyrë të përshtatshme, për shembull në një tavolinë
- ☐ Dhimbja më pengon të ngre pesha të rënda, por mund të menaxhoj pesha të lehta apo të mesme nëse ato janë të pozicionuara në mënyrë të përshtatshme
- ☐ Mund të ngre vetëm pesha shumë të lehta
- ☐ Nuk mund të ngre apo mbaj asgjë

SEKSIONI 4 – LEXIMI

- ☐ Mund të lexoj sa të dua pa pasur dhimbje në qafë
- ☐ Mund të lexoj sa të dua me dhimbje të lehtë në qafë
- ☐ Mund të lexoj sa të dua me dhimbje të moderuar në qafë
- ☐ Nuk mund të lexoj aq sa dua për shkak të dhimbjes së moderuar në qafë
- ☐ Mezi lexoj pak fare për shkak të dhimbjeve të forta në qafë
- ☐ Nuk lexoj dot fare

SEKSIONI 5 –DHIMBJA E KOKËS

- ☐ Nuk kam fare dhimbje koke
- ☐ Kam dhimbje koke të lehtë, e cila shfaqet rrallë
- ☐ Kam dhimbje koke të moderuar, e cila shfaqet rrallë
- ☐ Kam dhimbje koke të moderuar, e cila shfaqet shpesh
- ☐ Kam dhimbje koke të forta e te shpeshta
- ☐ Kam dhimbje koke pothuajse gjatë gjithë kohës

SEKSIONI 6 – PERQENDRIMI

- ☐ Mund të përqendrohem plotësisht kur dua, pa vështirësi
- ☐ Mund të përqendrohem plotësisht kur dua me pak vështirësi
- ☐ Kam pak vështirësi për t'u përqendruar
- ☐ Kam goxha vështirësi të përqendrohem kur dua
- ☐ E kam shumë të vështirë të përqendrohem kur dua
- ☐ Nuk mund të përqendrohem dot fare

SEKSIONI 7 – PUNA

- ☐ Mund të punoj sa dua
- ☐ Mund të bëj vetëm punën time të zakonshme, por jo më shumë
- ☐ Unë mund të bëj shumicën e punës sime të zakonshme, por jo më shumë
- ☐ Nuk mund të bëj punën time të zakonshme
- ☐ E kam të vështirë të mund të bëj ndonjë punë
- ☐ Nuk mund të bëj asnjë punë

SEKSIONI 8 – DREJTIMI I MAKINËS

- ☐ Mund ta ngas makinën time pa pasur dhimbje qafe
- ☐ Mund të ngas makinën time sa të dua me dhimbje të lehtë në qafë
- ☐ Mund të ngas makinën time sa të dua me dhimbje të moderuar në qafë
- ☐ Nuk mund ta ngas makinën time aq sa dua për shkak të dhimbjes së moderuar në qafë
- ☐ Vështirë se mund të ngas makinën për shkak të dhimbjeve të forta në qafë
- ☐ Nuk mund ta ngas makinën time fare

SEKSIONI 9 – GJUMI

- ☐ Nuk kam probleme me gjumin
- ☐ Kam pak probleme me gjumin (më pak se 1 orë pa gjumë)
- ☐ Kam pak probleme me gjumin (1-2 orë pa gjumë)
- ☐ Kam probleme të moderuara me gjumin (2-3 orë pa gjumë)
- ☐ Kam probleme të mëdha me gjumin (3-5 orë pa gjumë)
- ☐ E kam të vështirë gjumin (5-7 orë pa gjumë)

SEKSIONI 10 – RECREACIONI

- ☐ Mund të angazhohem në të gjitha aktivitetet e mia rekreative pa pasur dhimbje qafe fare
- ☐ Mund të angazhohem në të gjitha aktivitetet e mia rekreative, me pak dhimbje në qafë
- ☐ Mund të angazhohem në shumicën, por jo të gjitha aktivitetet e mia të zakonshme rekreative për shkak të dhimbjes në qafë
- ☐ Mund të angazhohem vetëm në disa nga aktivitetet e mia të zakonshme rekreative për shkak të dhimbjes në qafë
- ☐ Vështirë se mund të bëj ndonjë aktivitet rekreativ për shkak të dhimbjes në qafë
- ☐ Nuk mund të bëj fare aktivitete rekreative

EMRI I PACIENTIT ____ **DATA** ______________

PIKËT _________ [50]

NDI © Dr Howard Vernon, 1991. All Rights Reserved
